# Sublethal whole-body irradiation causes progressive premature frailty in mice

**DOI:** 10.1016/j.mad.2019.03.006

**Published:** 2019-06

**Authors:** Edward Fielder, Melanie Weigand, Julien Agneessens, Brigid Griffin, Craig Parker, Satomi Miwa, Thomas von Zglinicki

**Affiliations:** a Newcastle University Institute for Ageing and Institute for Cell and Molecular Biology, Campus for Ageing and Vitality, Newcastle University, Newcastle Upon Tyne, NE4 5PL, UK; b NIHR Newcastle Biomedical Research Centre, Institute of Neurosciences, Newcastle University, Campus for Ageing and Vitality, Newcastle Upon Tyne, NE4 5PL, UK

**Keywords:** FI, frailty Index, IR, irradiation, Frailty, Irradiation, Mice, Aging, Tumor survivor

## Abstract

There is an unmet need to develop and validate therapies that can treat or at least prevent premature therapy-induced frailty, multi-morbidity and mortality in long-term tumour survivors. In an approach to develop a first mouse model for therapy-induced long-term frailty, we irradiated male C57Bl/6 mice at 5–6 months of age sub-lethally with 3 × 3 Gy (whole body) and assessed subsequent frailty for up to 6 months using a Rockwood-type frailty index (FI). Frailty scorers were trained to obtain excellent inter- and intra-observer reproducibility. Irradiated mice developed progressive frailty approximately twice as fast as controls. This was premature frailty; it was phenotypically identical to that in non-irradiated mice at higher age. As expected, frailty was associated with decreased cognition and predicted mortality. In irradiated mice, frailty and neuromuscular performance, measured by Rotarod and Hanging Wire tests, were not associated with each other, probably because of long-term decreased body weights after irradiation. We conclude that progressive frailty following sub-lethal irradiation comprises a sensitive and easy to use test bed for interventions to stop premature ageing in long-term tumour survivors.

## Highlights

• Mice that survive irradiation develop progressive frailty.
• Irradiation-induced premature frailty is phenotypically equal to frailty in old animals.
• Premature frailty is associated with mortality and low cognition.
• Sub-lethal whole body irradiation may constitute a good mouse model to test efficiency of anti-ageing interventions.

## 1 Introduction

Improvements in cancer treatment have rendered many common cancers curable in a high proportion of patients. Although cancer remains a common disease, affecting an estimated 18 million of the world population in 2018, cancer-specific mortality has dropped sharply in the last few decades in developed countries. For example more than 70% of patients with breast cancer can now expect to live more than 10 years from diagnosis and many haematological and paediatric cancers have high cure rates (Bray et al., 2018). There is now greater awareness of health issues in long-term survivors, and in some fields the emphasis has started to shift towards efforts to improve the quality of survivorship after successful cancer treatment (Damlaj et al., 2019). The mainstays of adjuvant treatment i.e. radiotherapy and chemotherapy result in long-term morbidity in a wide range of organ systems, including cardiovascular, gastrointestinal, pulmonary, hepatic, musculoskeletal and neurological effects as well as enhanced mortality, together resembling accelerated ageing (Cupit-Link et al., 2017; Robison and Hudson, 2014). Specifically, widely used adjuvant therapies precipitate onset of frailty by about two decades of life prematurely in long-term survivors of many different cancer types (Arora et al., 2016; Ness et al., 2015, 2013). At present, the only approach to postpone or lessen this therapy-induced frailty is lifestyle counselling; there are no therapies available. To develop and validate those, mouse models of therapy-induced frailty are urgently needed.

The concept of frailty has been developed to describe the conditions of aged people with increased vulnerability to adverse health outcomes and is considered to be associated with a major loss of capacity to maintain tissue homeostasis and regeneration. It is characterized by a state of age-related biological vulnerability to stressors and decreased physiological reserves with alterations in energy metabolism, decreased skeletal muscle mass and quality, and altered hormonal and inflammatory functions (for review see (Mohler et al., 2014)). A consensus paper defined frailty as “a medical syndrome with multiple causes and contributors that is characterized by diminished strength and endurance, and reduced physiologic function that increase an individual’s vulnerability for developing increased dependency and/or death” (Morley et al., 2013).

A number of frailty scales have been developed to operationalize the assessment of human frailty. The two major frailty models that have been more extensively validated are the “Fried frailty phenotype” and the “Rockwood frailty index (FI)”. Fried’s frailty phenotype defines frailty as a distinct clinical syndrome meeting three or more of five phenotypic criteria: weakness, slowness, low level of physical activity, self-reported exhaustion, and unintentional weight loss (Fried et al., 2001). In contrast, the FI defined frailty by the accumulation of deficits in multiple domains (Rockwood et al., 2005). Deficiencies in more than 70 parameters relevant to everyday activities, also comprising physiological problems, mental capabilities, concomitant features of co-morbidities etc. have been included in the construction of various Rockwood frailty index scales. Although multi-deficiency and multi-morbidity is an essential part of the frailty index, the specific number and types of deficiencies included have only a minor impact on the categorization of frailty (Song et al., 2010). Moreover, additional parameters in excess of about 30 do not seem to increase the power of the index greatly. While the Fried and Rockwood concepts are overlapping and both scales are able to predict mortality, sensitivity and specificity regarding the classification of individuals as frail/non-frail are somewhat different between the scoring systems (Collerton et al., 2012; Ravindrarajah et al., 2013; Theou et al., 2013).

In recent years, both frailty models have been reverse translated into mice (see (von Zglinicki et al., 2016) for review). Rockwood-type frailty indices for mice have bene constructed based on either observational scoring (Whitehead et al., 2014) or quantitative measurements (Antoch et al., 2017), while a Fried-type frailty syndrome for mice is usually constructed from quantitative measurements (Baumann et al., 2018; Martinez de Toda et al., 2018). The reliability (longitudinal and inter-observer) of the FI scores has been validated (Feridooni et al., 2015; Kane et al., 2015) and all scales were able to predict mortality in mice (Antoch et al., 2017; Baumann et al., 2018; Martinez de Toda et al., 2018; Rockwood et al., 2017). Both the scored (Kane et al., 2016) and the quantitatively based (Antoch et al., 2017) FI have been shown to be sensitive to longevity-modulating interventions (caloric restriction, high-fat feeding, treatment with resveratrol or mTOR inhibitor) at least in some cohorts of mice.

There is yet little data available on long-term effects of either chemo- or radiotherapy on frailty in mice. Available mouse models of radiation- or chemotherapy-induced fatigue cover only some aspects of frailty and importantly are generally only evaluated over short periods, covering essentially only the period of acute radio- or chemotoxicity (Demaria et al., 2017; Dougherty et al., 2019; Renner et al., 2016; Wolff et al., 2017). In one paper, running wheel activity after localized irradiation (single leg) has been shown to be compromised for up to seven months (Zhu et al., 2015), but further domains of frailty were not assessed. Assessing frailty according to Whitehead (Whitehead et al., 2014) longitudinally, we show here that sub-lethal whole-body irradiation induces progressive premature frailty in mice. Radiation-induced frailty predicts severe morbidity precipitating mortality and is associated with cognitive decline. Associations between frailty and neuromuscular capability as measured by performance in rotarod and hanging wire tests are complicated by irradiation-mediated body weight decrease. The data suggest this as a simple, relevant test bed for interventions aimed at preventing or relieving cancer therapy-induced premature frailty and ageing.

## 2 Material and methods

### 2.1 Mice

Male C57Bl/6 mice were bought past weaning from Charles River and were maintained in groups of six littermates per cage as described (Cameron et al., 2012). Most mice were fed standard pelleted food (CRM-P formulation rodent diet, SDS Diets), but some mice were either gavage-fed for two weeks or received soaked food (same as above) from one month post-IR. The work was licensed by the UK Home Office (PB048F3A0) and complied with the guiding principles for the care and use of laboratory animals.

### 2.2 Irradiation

At 5–6 months of age, mice were sub-lethally irradiated (NDT 320 or X-RAD225, 225 kV) with 3 Gy of X-ray irradiation, 3 times, with 2 days recovery time between doses. 2 days prior to IR, and for 14 days post-IR, mice received 1% Baytril solution (Broad-spectrum antibiotic) in drinking water.

### 2.3 Frailty index

Frailty was assessed as a 30-parameter index based on (Whitehead et al., 2014). In comparison to (Whitehead et al., 2014), we did not test the menace reflex in order to reduce the stress to the animals. Moreover, grip strength was measured using BIO-GS3 BIOSEP and the average of 3 measurements was compared to reference values from sex-matched adult animals. Further minor modifications in the operationalisation are indicated in Supplementary Tab. S1.

### 2.4 Neuromuscular tests

Hanging Wire: Animals were allowed to grip the middle of a suspended plastic wire (diameter 2 mm, and 30 cm in length) with their forelimbs and let to hang. Test duration was 60 s and success was measured as a mouse hanging through the whole test, getting all 4 limbs onto the wire (time taken recorded), or reaching the end of the wire (time taken recorded). Failure was measured as a mouse falling before 60 s. Mice were given up to 3 trials to succeed (spaced by 5 min). The wire was suspended 30 cm above a landing pad (20 cm depth of soft fluffed bedding, and with a paper cover), which proved high enough to discourage deliberate dropping but reduced the impact from falling.

Rotarod (LE8200 BIOSEP) was used to assess muscle function. Mice were placed on the rod at an initial 4 rpm, and rotation accelerated by 7.2 rpm per minute. Maximum velocity (rpm), time on rod (sec) and total distance (m) were recorded. Mice were tested 3 times per day, for up to 3 days, with approximately 15 min intervals between trials.

### 2.5 Cognition

Two protocols were used with separate Y-Mazes: Forced Alternation, and Spontaneous Alternation. For forced alternation, the Y-maze was made from white plastic. Each arm was 40 cm long, 8 cm wide, and 15 cm high with a curved lip. The end of each arm was marked with a black icon (+,▪, o). The home cage was moved into the test room for one hour prior to testing to allow mice to acclimatise. Experiments were performed in 80 lx light to allow for video recording. During acquisition, one of the arms (▪ or o) was blocked off by a white barrier. The mouse was placed in the home arm (+) and given 10 min to explore the two open arms of the maze (home and familiar arms). After this, the mouse was returned to the home cage for a 1 h inter-trial period. The maze was cleaned thoroughly with 70% EtOH between trials to remove odours. Up to 5 mice could be tested at a time. After 1 h, the mouse was placed in the home arm, with the blocked off arm (novel arm) opened and all 3 arms (home, familiar and novel arms) open to explore for 5 min. If the mouse climbed out of the maze, it was returned to the abandoned spot. Time taken for mice to enter the novel arm was recorded.

The Spontaneous Alternation Y-maze was performed in a separate Y-maze, made of dark grey plastic, each arm 40 cm long, 5 cm wide and 10 cm high. Mice were placed in arm 1, and order of arm entry was manually recorded for 5 min in low light conditions (˜1 lx). A spontaneous alternation is defined as a mouse entering a different arm of the maze in each 3 consecutive entries, spontaneous alternation frequency is calculated as the number of spontaneous alternations, divided by total number of arm entries -2.

### 2.6 Statistics

Non-normally distributed data were presented as box-and-whisker plots with boxes indicating median and upper/lower quartiles, whiskers indicating upper/lower percentiles and outliers shown. These data were compared by Mann-Whitney Rank Sum test. Multiple groups were compared by ANOVA or, if the data failed an equal variance test, by ANOVA on ranks, followed by post-hoc tests (Holm-Sidak or Dunn’s). Survival was assessed by Kaplan-Meier Analysis, comparing groups by Log-Rank test. All correlations were assumed as linear and assessed by Pearson’s statistics.

## 3 Results and discussion

### 3.1 Establishing reproducibility in FI scoring

**Fig. 1:**
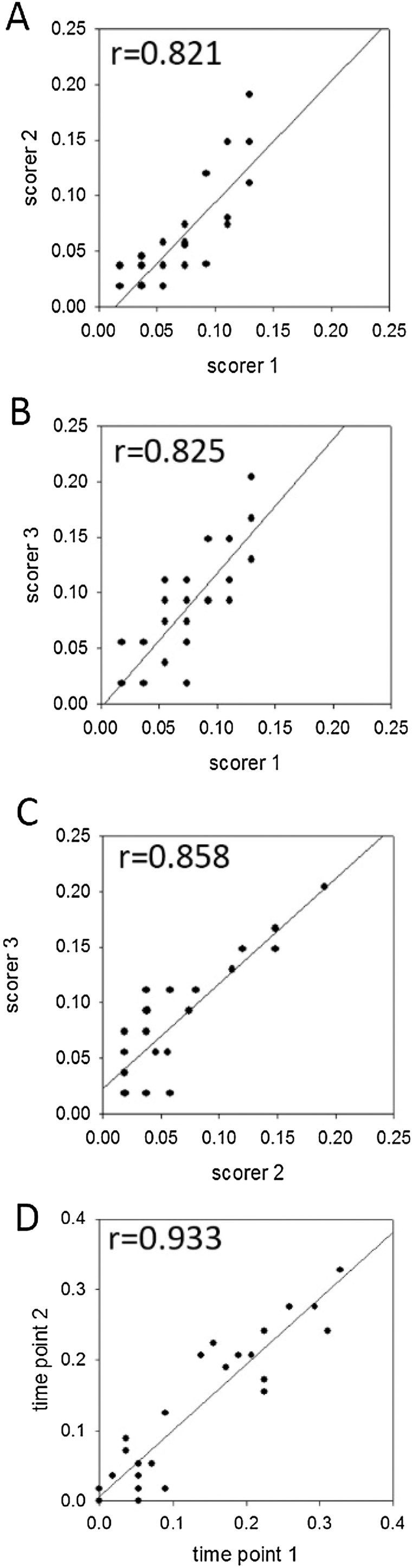
**Inter- and intra-scorer reproducibility of FI scores. A–C)** FI scores for individual mice obtained by three different observers independently. Mice were 7 months old, half of them had been irradiated at 5 months of age. **D)** FI scores obtained by the same observer longitudinally at time points 2 weeks apart. Data are from two groups of 12 mice each at 5 and 22 months of age. Pearsons’ linear correlation coefficients r are shown.

To enable meaningful assessment of FI scores during longitudinal ageing studies, both intra- and inter-scorer reproducibility has to be established. We trained three lab members (a postdoc, a PhD student and a technician, all with previous experience in mouse husbandry, but only the post-doc with previous experience in frailty assessment) to perform frailty assessments interchangeably based on the same operationalisation. Over a course of six weeks, weekly frailty assessments were performed by the three scorers as a group, during which the operationalisation of the scoring was discussed and modified towards a finally agreed version. Afterwards, each assessor scored a group of 20 mice independently without interaction with the others. Post-hoc analysis showed very good correlations between scores from all three observers (Fig. 1A–C). Another group of mice was scored by the same observer twice over the course of 2 weeks. The second scoring was done without access to the results of the first. Both data sets were in excellent accordance (Fig. 1D).

The inter-rater reliability achieved by this training period (Fig. 1) exceeded those reported by two independent groups previously (Feridooni et al., 2015; Kane et al., 2015). To ensure consistence with ongoing and future studies, new observers will only be added to the scorer pool after being similarly trained by at last one member of the original group and after establishing reproducibility.

### 3.2 Whole body irradiation causes premature frailty

**Fig. 2:**
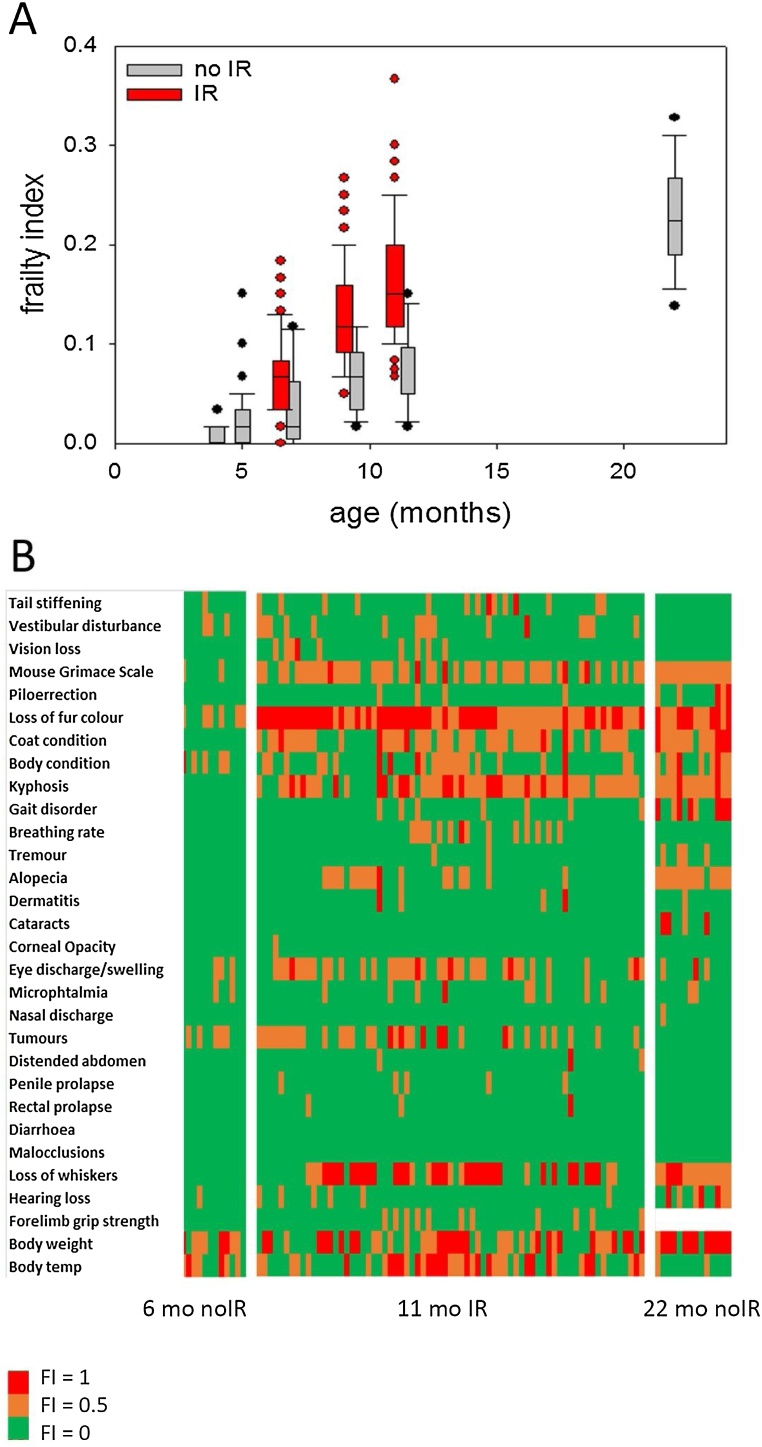
**Whole-body IR causes premature frailty in mice. A)** C57Bl/6 male mice were irradiated or sham-irradiated at 5–6 months of age and frailty was scored at the indicated time points. Scoring was done longitudinally with the exception of the 22 months old animals (n = 15). Other data are from 87 irradiated and 12 sham-irradiated mice. *** p < 0.001, Mann-Whitney Rank Sum test. **B)** Irradiation-induced frailty domains are similar to those found in old mice. Heatmap shows scores for all 30 frailty domains in 12 young sham-irradiated mice (left), 71 irradiated mice at 11 months of age (middle) and 14 22 months old non-irradiated mice. Scores are 0 (green), 0.5 (yellow) or 1 (red).

For this study, 88 mice were irradiated with 3 times 3 Gy in four cohorts at 5–6 months of age (between days 140 and 181). One mouse had to be culled due to acute radiation sickness before the first frailty assessment. In all other mice, indicators of acute radiation thickness (mild to moderate weight loss, white toes) abated within a month and post-IR frailty was scored about every two months. Mortality was progressively high (see below): 2 mice had to culled between first and second frailty assessment, and 10 more before the third, mostly due to tumours (thymoma). Frailty scores in the irradiated mice were compared to a group of 12 mice that were sham-irradiated at 6 months of age as well as to a group of 15 non-irradiated mice at 22 months of age (Fig. 2A). Frailty scores assessed in the latter group by us were quantitatively equal to those described by others in the same strain and age (Kane et al., 2016; Rockwood et al., 2017). Already at 2 months post IR, the FI in the irradiated mice was higher than in the control, and it increased progressively at about twice the control rate, reaching a level similar to that of 22 months old non-irradiated animals already at 11 months of age (Fig. 2A).

A comparison of the individual domains of the FI between 11 months old irradiated and 22 months old non-irradiated mice revealed deficiencies in exactly the same domains in both groups of mice (Fig. 2B). Frailty precipitated by IR develops faster but is phenotypically equal to frailty associated with the ‘normal’ ageing process. In other words, whole-body irradiation causes progressive premature frailty. This is important because it implies that irradiated mice constitute a valid model to test interventions aimed at preventing or curing age-related frailty but at about half the time and costs that would normally be necessary.

### 3.3 Radiation-induced frailty predicts mortality

**Fig. 3:**
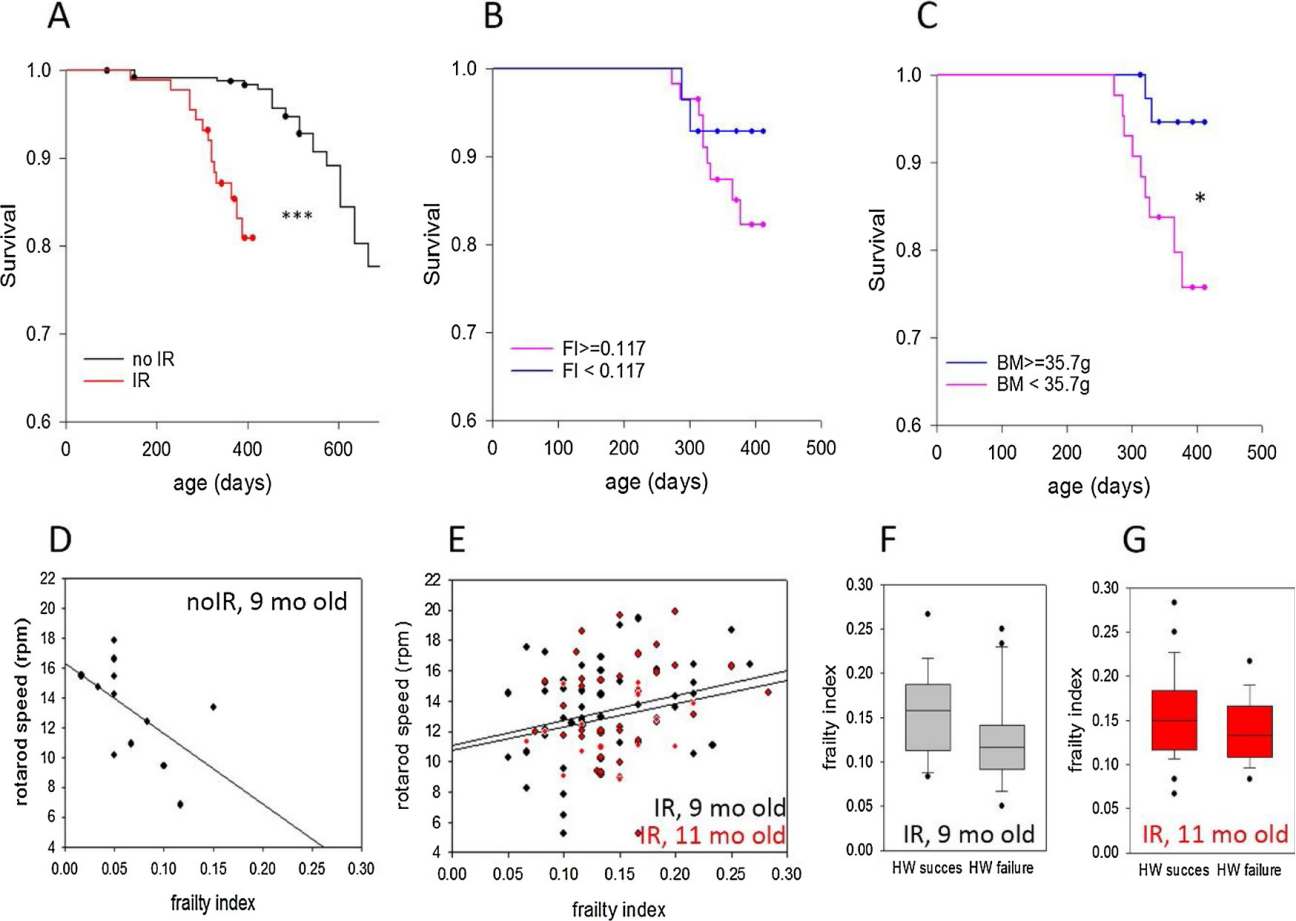
**Radiation-induced premature frailty predicts mortality but not loss of neuromuscular coordination. A)** Kaplan-Meyer survival curves for irradiated vs non-irradiated male C57Bl/6 mice. Data are from 87 irradiated and 261 non-irradiated mice. Irradiation was started between 140 and 181 days of age. p < 0.001. **B)** Survival of irradiated mice split according to their FI median value (0.117) measured at about 4 months post IR. **C)** Survival of irradiated mice split according to their body mass median value (35.7 g) measured at about 4 months post IR. **D)** Association between FI and maximum rotarod speed in sham-irradiated mice at 9 months of age. p = 0.008. **E)** Association between FI and maximum rotarod speed in irradiated mice at 9 months of age (4 months past IR, black, p = 0.055) and at 11 months of age (red, p = 0.114). **F)** FI in irradiated mice at about 4 months past IR that succeeded (left) or failed (right) the hanging wire test. p = 0.059. **G)** FI in irradiated mice at about 6 months past IR that succeeded (left) or failed (right) the hanging wire test. p = 0.258.

The FI predicts mortality risk in both humans and mice (Rockwood et al., 2017). Accordingly, in our cohort, irradiated mice showed not only premature frailty but also enhanced mortality (Fig. 3A). It should however be stressed that the concept of mortality in mice is very different from humans: mice are being humanely killed if they show signs of significant distress such that ‘natural’ death occurs only as a very rare exception. Therefore, mortality in mice is much more closely associated with morbidity than in humans. From our cohort of 88 irradiated mice, 15 had to be culled during the observation period of 412 days. Of these, one mouse as culled because of severe acute radiation sickness within days after irradiation and was excluded from Fig. 3A. 3 animals were culled because of swollen, inflamed legs, probably caused by fight wounds and one mouse developed a sore penis and prolapse. The vast majority (11 out of 15) of mice presented with tumours, mostly thymomas.

When irradiated mice were stratified according to the median frailty index measured at 4 months post-IR, a suggestive albeit not significant difference in survival was seen (Fig. 3B). However, body mass, measured at the same time point as this second post-IR frailty assessment, was a significant predictor of survival (Fig. 3C). This difference in predictive power might be due to the fact that follow-up time in our cohorts was relatively short (median follow-up age 251 days, maximum age 412 days). Low body mass and especially fast weight loss is a well-established predictor of immanent death in both humans (Fried et al., 2001) and mice (Jurk et al., 2014). It might be possible that the FI would gain better predictive power over a longer prospective period.

We next examined the associations between frailty index and neuromuscular coordination, measured by Rotarod and Hanging Wire tests that were performed in parallel to the frailty assessments. In sham-irradiated mice, the expected inverse association between frailty and maximum obtainable speed on a Rotarod was seen (Fig. 3D). Unexpectedly, irradiated mice, despite being more frail (see Fig. 2A), did not show lower performance in either test at any time (data not shown). The association between FI and Rotarod performance in irradiated mice was actually borderline positive at 4 months post-IR and did not become inverse even at 6 months post-IR (Fig. 3E). Similar results were obtained when time on the rod or distance travelled were considered (data not shown). Moreover, success or failure in the Hanging Wire test was not dependent on frailty levels in either 9 months old (Fig. 3F) or 11 months old (Fig. 3G) irradiated mice. If anything, there was a tendency for an association of lower frailty levels with test failure in the younger mice (Fig. 3F), again contrary to expectations. Similarly, (Kane et al., 2016) did not find a correlation between frailty index and neuromuscular performance in subgroups of their calorie restricted or resveratrol-treated mice. Associations between body mass and functional performance measures were not reported in this study.

Body mass is a significant negative determinant of performance in neuromuscular tests (Graber et al., 2013) and remained lower in irradiated mice over the whole post-IR observation period (suppl. Fig. S1). Our results confirm low body mass as a major predictor of good physical performance and low frailty at younger biological age, i.e. in sham-irradiated mice (suppl. Fig. S2A, B). However, at more advanced biological age, low body mass becomes a predictor of increased frailty and mortality risk in humans (Fried et al., 2001) and mice (Jurk et al., 2014). Accordingly, in the irradiated mice body weight still negatively predicts Rotarod performance at 9 months of age but the correlation is lost at 11 months (Suppl. Fig. S2C). Similarly, low body mass predicts success in the hanging wire test at the earlier but not the later age (suppl. Fig. S2E, F). The association of body mass with frailty is lost in irradiated mice already at 9 months of age (suppl. Fig. S2D). Together, these data show that body mass is a potentially major but complex confounder of the association between frailty and neuromuscular performance. Given that large weight differences between sham-treated and irradiated mice persist for many months after irradiation (suppl. Fig. S1), we would argue against normalising performance measures by body weight as this might introduce apparent but artificial differences in performance.

### 3.4 Radiation-induced frailty is associated with decreased cognition

**Fig. 4:**
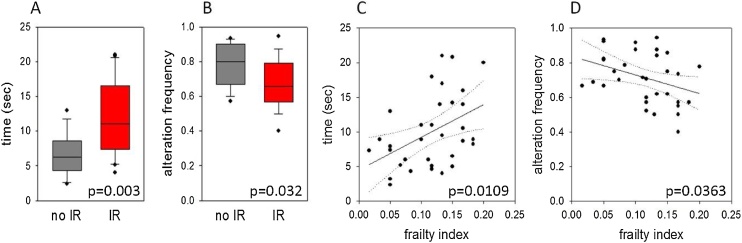
**Frailty is associated with low cognition.** Cognition was assessed in 12 sham-irradiated control mice (no IR) and in 22 irradiated mice (IR) at11 months of age using a Y-maze by either time to enter the novel arm (forced alternation, **A)** or alternation frequency in a spontaneous alternation setting **(B)**. **C)** Correlation between FI and time to enter the novel arm. Regression line and 95% prediction intervals are shown. P = 0.0109. **D)** Correlation between FI and alternation frequency. P = 0.0363.

We tested cognition in parallel with the last frailty assessment by two versions of the Y maze test, measuring i) the time it took a mouse to enter the novel arm in a forced alternation experiment (Fig. 4A) and ii) the alternation frequency in a spontaneous alternation set-up (Fig. 4B). In both tests, irradiated mice performed worse than sham-treated ones (Fig. 4A, B). The FI at the time of cognitive assessment predicted these differences in cognition at single animal level (Fig. 4C, D). However, FI values assessed at an earlier time point (i.e. at 4 months post-IR) did not prospectively predict cognitive performance (data not shown).

Y maze performance may be compromised by vision impairment or loss of whiskers, both of which were absent in the sham-irradiated mice but were found in some of the irradiated animals. Thus, lower performance in the maze post irradiation might simply reflect these sensory organ impairments rather than decreased cognition. To address this possibility, we separated the Y maze results in the irradiated mice according to their whisker and vision status (suppl. Fig. S3). Irradiated mice still showed delayed entry into the novel arm even when they retained whiskers (suppl. Fig. S3A) and with intact vision (suppl. Fig. S3C). However, the reduced ability of irradiated mice to discriminate between maze arms in a spontaneous alternation experiment was no longer significant when physically impaired mice were separated out (suppl. Fig. S3B, D). Correlations between FI and Y maze performance remained weakly significant for mice that retained their whiskers (p = 0.047 for FI vs time; p = 0.096 for FI vs alternation frequency). For mice with unimpaired vision, the correlation between FI and time remained significant (p = 0.007), but FI and alternation frequency were no longer correlated (p = 0.132). Together, these data show that loss of whiskers or vision impairment contribute to lower performance of irradiated mice in the Y maze to some extent. However, irradiated mice without such sensory organ impairments also performed worse in at least some of the tests, suggesting an independent effect of irradiation on cognition.

### 3.5 Conclusions

We show here that fractionated, sub-lethal irradiation causes premature frailty, cognitive decline and premature mortality, mostly associated with early tumour development. Already within two months after irradiation, the frailty index according to (Whitehead et al., 2014) is significantly enhanced and increases progressively with time. This index is easy, fast and cheap to assess, can be measured longitudinally and is highly reproducible following a short training programme. It provides a simple model for testing and pre-clinical validation of interventions aimed to treat therapy-induced premature ageing in long-term cancer survivors.

Limitations of our study include that it has been done in one strain of mice only and only in males. It will now be important to address the question whether localized irradiation, simulating more closely a cancer therapeutic situation, also causes progressive frailty in mice.

## Funding

This work was supported by Cancer Research UK (Pioneer grant C12161/A24009 to TvZ) and by Innovate UK (KTP No 11272 to SM and TvZ). The sponsors had no role in study design; in the collection, analysis and interpretation of data; in the writing of the report; and in the decision to submit the article for publication.

## References

[bib0005] Antoch M.P., Wrobel M., Kuropatwinski K.K., Gitlin I., Leonova K.I., Toshkov I., Gleiberman A.S., Hutson A.D., Chernova O.B., Gudkov A.V. (2017). Physiological frailty index (PFI): quantitative in-life estimate of individual biological age in mice. Aging (Albany N. Y.).

[bib0010] Arora M., Sun C.L., Ness K.K., Teh J.B., Wu J., Francisco L., Armenian S.H., Schad A., Namdar G., Bosworth A., Kuo L., Weisdorf D.J., Forman S.J., Bhatia S. (2016). Physiologic frailty in nonelderly hematopoietic cell transplantation patients: results from the bone marrow transplant survivor study. JAMA Oncol..

[bib0015] Baumann C.W., Kwak D., Thompson L.V. (2018). Assessing onset, prevalence and survival in mice using a frailty phenotype. Aging (Albany N. Y.).

[bib0020] Bray F., Ferlay J., Soerjomataram I., Siegel R.L., Torre L.A., Jemal A. (2018). Global cancer statistics 2018: GLOBOCAN estimates of incidence and mortality worldwide for 36 cancers in 185 countries. CA Cancer J. Clin..

[bib0025] Cameron K.M., Miwa S., Walker C., von Zglinicki T. (2012). Male mice retain a metabolic memory of improved glucose tolerance induced during adult onset, short-term dietary restriction. Longev. Healthspan.

[bib0030] Collerton J., Martin-Ruiz C., Davies K., Hilkens C.M., Isaacs J., Kolenda C., Parker C., Dunn M., Catt M., Jagger C., von Zglinicki T., Kirkwood T.B. (2012). Frailty and the role of inflammation, immunosenescence and cellular ageing in the very old: cross-sectional findings from the Newcastle 85+ Study. Mech. Ageing Dev..

[bib0035] Cupit-Link M.C., Kirkland J.L., Ness K.K., Armstrong G.T., Tchkonia T., LeBrasseur N.K., Armenian S.H., Ruddy K.J., Hashmi S.K. (2017). Biology of premature ageing in survivors of cancer. ESMO Open.

[bib0040] Damlaj M., El Fakih R., Hashmi S.K. (2019). Evolution of survivorship in lymphoma, myeloma and leukemia: metamorphosis of the field into long term follow-up care. Blood Rev..

[bib0045] Demaria M., O’Leary M.N., Chang J., Shao L., Liu S., Alimirah F., Koenig K., Le C., Mitin N., Deal A.M., Alston S., Academia E.C., Kilmarx S., Valdovinos A., Wang B., de Bruin A., Kennedy B.K., Melov S., Zhou D., Sharpless N.E., Muss H., Campisi J. (2017). Cellular senescence promotes adverse effects of chemotherapy and cancer relapse. Cancer Discov..

[bib0050] Dougherty J.P., Springer D.A., Cullen M.J., Gershengorn M.C. (2019). Evaluation of the effects of chemotherapy-induced fatigue and pharmacological interventions in multiple mouse behavioral assays. Behav. Brain Res..

[bib0055] Feridooni H.A., Sun M.H., Rockwood K., Howlett S.E. (2015). Reliability of a frailty index based on the clinical assessment of health deficits in male C57BL/6J mice. J. Gerontol. A Biol. Sci. Med. Sci..

[bib0060] Fried L.P., Tangen C.M., Walston J., Newman A.B., Hirsch C., Gottdiener J., Seeman T., Tracy R., Kop W.J., Burke G., McBurnie M.A., Cardiovascular Health Study Collaborative Research G. (2001). Frailty in older adults: evidence for a phenotype. J. Gerontol. A Biol. Sci. Med. Sci..

[bib0065] Graber T.G., Ferguson-Stegall L., Kim J.H., Thompson L.V. (2013). C57BL/6 neuromuscular healthspan scoring system. J. Gerontol. A Biol. Sci. Med. Sci..

[bib0070] Jurk D., Wilson C., Passos J.F., Oakley F., Correia-Melo C., Greaves L., Saretzki G., Fox C., Lawless C., Anderson R., Hewitt G., Pender S.L., Fullard N., Nelson G., Mann J., van de Sluis B., Mann D.A., von Zglinicki T. (2014). Chronic inflammation induces telomere dysfunction and accelerates ageing in mice. Nat. Commun..

[bib0075] Kane A.E., Hilmer S.N., Huizer-Pajkos A., Mach J., Nines D., Boyer D., Gavin K., Mitchell S.J., de Cabo R. (2015). Factors that impact on interrater reliability of the mouse clinical frailty index. J. Gerontol. A Biol. Sci. Med. Sci..

[bib0080] Kane A.E., Hilmer S.N., Boyer D., Gavin K., Nines D., Howlett S.E., de Cabo R., Mitchell S.J. (2016). Impact of longevity interventions on a validated mouse clinical frailty index. J. Gerontol. A Biol. Sci. Med. Sci..

[bib0085] Martinez de Toda I., Garrido A., Vida C., Gomez-Cabrera M.C., Vina J., De la Fuente M. (2018). Frailty quantified by the "Valencia score" as a potential predictor of lifespan in mice. J. Gerontol. A Biol. Sci. Med. Sci..

[bib0090] Mohler M.J., Fain M.J., Wertheimer A.M., Najafi B., Nikolich-Zugich J. (2014). The Frailty syndrome: clinical measurements and basic underpinnings in humans and animals. Exp. Gerontol..

[bib0095] Morley J.E., Vellas B., van Kan G.A., Anker S.D., Bauer J.M., Bernabei R., Cesari M., Chumlea W.C., Doehner W., Evans J., Fried L.P., Guralnik J.M., Katz P.R., Malmstrom T.K., McCarter R.J., Gutierrez Robledo L.M., Rockwood K., von Haehling S., Vandewoude M.F., Walston J. (2013). Frailty consensus: a call to action. J. Am. Med. Dir. Assoc..

[bib0100] Ness K.K., Krull K.R., Jones K.E., Mulrooney D.A., Armstrong G.T., Green D.M., Chemaitilly W., Smith W.A., Wilson C.L., Sklar C.A., Shelton K., Srivastava D.K., Ali S., Robison L.L., Hudson M.M. (2013). Physiologic frailty as a sign of accelerated aging among adult survivors of childhood cancer: a report from the St Jude Lifetime cohort study. J. Clin. Oncol..

[bib0105] Ness K.K., Armstrong G.T., Kundu M., Wilson C.L., Tchkonia T., Kirkland J.L. (2015). Frailty in childhood cancer survivors. Cancer.

[bib0110] Ravindrarajah R., Lee D.M., Pye S.R., Gielen E., Boonen S., Vanderschueren D., Pendleton N., Finn J.D., Tajar A., O’Connell M.D., Rockwood K., Bartfai G., Casanueva F.F., Forti G., Giwercman A., Han T.S., Huhtaniemi I.T., Kula K., Lean M.E., Punab M., Wu F.C., O’Neill T.W., European Male Aging Study, G (2013). The ability of three different models of frailty to predict all-cause mortality: results from the European Male Aging Study (EMAS). Arch. Gerontol. Geriatr..

[bib0115] Renner M., Feng R., Springer D., Chen M.K., Ntamack A., Espina A., Saligan L.N. (2016). A murine model of peripheral irradiation-induced fatigue. Behav. Brain Res..

[bib0120] Robison L.L., Hudson M.M. (2014). Survivors of childhood and adolescent cancer: life-long risks and responsibilities. Nat. Rev. Cancer.

[bib0125] Rockwood K., Song X., MacKnight C., Bergman H., Hogan D.B., McDowell I., Mitnitski A. (2005). A global clinical measure of fitness and frailty in elderly people. CMAJ.

[bib0130] Rockwood K., Blodgett J.M., Theou O., Sun M.H., Feridooni H.A., Mitnitski A., Rose R.A., Godin J., Gregson E., Howlett S.E. (2017). A frailty index based on deficit accumulation quantifies mortality risk in humans and in mice. Sci. Rep..

[bib0135] Song X., Mitnitski A., Rockwood K. (2010). Prevalence and 10-year outcomes of frailty in older adults in relation to deficit accumulation. J. Am. Geriatr. Soc..

[bib0140] Theou O., Brothers T.D., Mitnitski A., Rockwood K. (2013). Operationalization of frailty using eight commonly used scales and comparison of their ability to predict all-cause mortality. J. Am. Geriatr. Soc..

[bib0145] von Zglinicki T., Varela Nieto I., Brites D., Karagianni N., Ortolano S., Georgopoulos S., Cardoso A.L., Novella S., Lepperdinger G., Trendelenburg A.U., van Os R. (2016). Frailty in mouse ageing: a conceptual approach. Mech. Ageing Dev..

[bib0150] Whitehead J.C., Hildebrand B.A., Sun M., Rockwood M.R., Rose R.A., Rockwood K., Howlett S.E. (2014). A clinical frailty index in aging mice: comparisons with frailty index data in humans. J. Gerontol. A Biol. Sci. Med. Sci..

[bib0155] Wolff B.S., Renner M.A., Springer D.A., Saligan L.N. (2017). A mouse model of fatigue induced by peripheral irradiation. J. Vis. Exp..

[bib0160] Zhu Y., Tchkonia T., Pirtskhalava T., Gower A.C., Ding H., Giorgadze N., Palmer A.K., Ikeno Y., Hubbard G.B., Lenburg M., O’Hara S.P., LaRusso N.F., Miller J.D., Roos C.M., Verzosa G.C., LeBrasseur N.K., Wren J.D., Farr J.N., Khosla S., Stout M.B., McGowan S.J., Fuhrmann-Stroissnigg H., Gurkar A.U., Zhao J., Colangelo D., Dorronsoro A., Ling Y.Y., Barghouthy A.S., Navarro D.C., Sano T., Robbins P.D., Niedernhofer L.J., Kirkland J.L. (2015). The Achilles’ heel of senescent cells: from transcriptome to senolytic drugs. Aging Cell.

